# The Colon Health and Life-Long Exercise Change trial: a randomized trial of the National Cancer Institute of Canada Clinical Trials Group

**DOI:** 10.3747/co.v15i6.378

**Published:** 2008-12

**Authors:** K.S. Courneya, C.M. Booth, S. Gill, P. O’Brien, J. Vardy, C.M. Friedenreich, H.J. Au, M.D. Brundage, D. Tu, H. Dhillon, R.M. Meyer

**Affiliations:** * University of Alberta, Edmonton, AB; † National Cancer Institute of Canada Clinical Trials Group, Queen’s University, Kingston, ON; ‡ University of British Columbia, Vancouver, BC; § University of Sydney, Sydney, Australia; || Alberta Cancer Board, Calgary, AB; # Cross Cancer Institute, Edmonton, AB

**Keywords:** Behavioural oncology, cancer survivor, disease-free survival, exercise, lifestyle, physical activity, quality of life, survivorship

## Abstract

**Background:**

Observational studies indicate that physical activity (pa) is strongly associated with improved disease outcomes in colon cancer survivors, but a randomized controlled trial is needed to determine whether the association is causal and whether new policies to promote exercise are justified.

**Purpose:**

The co.21 Colon Health and Life-Long Exercise Change (challenge) trial undertaken by the National Cancer Institute of Canada Clinical Trials Group (ncic ctg) is designed to determine the effects of a structured pa intervention on outcomes for survivors of high-risk stage ii or iii colon cancer who have completed adjuvant therapy within the previous 2–6 months.

**Methods:**

Trial participants (*n* = 962) will be stratified by centre, disease stage, body mass index, and performance status, and will be randomly assigned to a structured pa intervention or to general health education materials. The pa intervention will consist of a behavioural support program and supervised pa sessions delivered over a 3-year period, beginning with regular face-to-face sessions and tapering to less frequent face-to-face or telephone sessions. The primary endpoint is disease-free survival. Important secondary endpoints include multiple patient-reported outcomes, objective physical functioning, biologic correlative markers, and an economic analysis.

**Summary:**

Cancer survivors and cancer care professionals are interested in the potential role of PA to improve multiple disease-related outcomes, but a randomized controlled trial is needed to provide compelling evidence to justify changes in health care policies and practice.

## 1. INTRODUCTION

Since the 1990s, a 6-month course of adjuvant chemotherapy has been the standard of care for patients with resected high-risk stage ii or iii colon cancer 1. Despite recent advances in adjuvant therapy, a substantial proportion of such patients still experience disease relapse and premature death 2. Recently published observational data suggest that physical activity (pa) is associated with a reduced risk of disease relapse and premature death in these colon cancer survivors.

In 2006, Meyerhardt *et al.* 3 reported results of a prospective observational study of 832 patients with stage iii colon cancer enrolled in a randomized adjuvant chemotherapy trial and followed for a median of 3.8 years from trial entry. In that trial, pa was self-reported approximately 6 months after completion of chemotherapy and was quantified as weekly metabolic equivalent task (met)–hours (for example, walking briskly is approximately 4.0 mets). Categories of pa were predefined in terms of these met–hours: fewer than 3 (referent), 3–8.9, 9–17.9, 18–26.9, and more than 27. Analyses adjusted for known prognostic factors, including body mass index (bmi), indicated that higher levels of pa were associated with superior disease-free (dfs), recurrence-free, and overall survival. The 3-year dfs was 75.1% in patients who exercised for fewer than 18 met–hours weekly as compared with 84.5% in patients who exercised for more than 18 met–hours weekly [hazard ratio (hr): 0.57; 95% confidence interval (ci): 0.39 to 0.85].

In a second article, Meyerhardt *et al.* 4 reported results of a prospective observational study of 573 women from the Nurses’ Health Study diagnosed with stages i–iii colorectal cancer. Self-reported leisure-time pa before diagnosis and 1–4 years post-diagnosis was assessed, and analyses were again adjusted for known prognostic factors, including bmi. An inverse relationship was observed between the amount of pa post-diagnosis and the risk of both colorectal cancer–specific and overall mortality. Specifically, as compared with women exercising for fewer than 3 met–hours weekly, the risk of colorectal cancer–specific mortality declined in successive groups performing more exercise: the hr was 0.92 (95% ci: 0.50 to 1.69) in the 3–8.9 met–hours weekly group, 0.57 (95% ci: 0.27 to 1.20) in the 9–17.9 met–hours weekly group, and 0.39 (95% ci: 0.18 to 0.82) in the more than 18 met–hours weekly group. Risk of overall mortality was similarly reduced. Furthermore, change in pa from pre- to post-diagnosis was also predictive of outcome. Compared with women who did not change their pa, women who increased their pa by at least 1 predefined met category—for example, from fewer than 3 met–hours to 3–8.9 met–hours—between their pre-diagnostic and post-treatment assessments experienced improved outcomes; women whose exercise levels decreased by at least 1 predefined met category had poorer outcomes. Further analysis of these data suggested that no additional benefit accrued with an increase in pa post-diagnosis in women who were already achieving at least 9 met–hours weekly before diagnosis (that is, those who were roughly already meeting current public health guidelines).

Several plausible biologic mechanisms could account for an association between pa and colon cancer outcomes, including metabolic consequences of obesity; decreased gastrointestinal transit time; decreased levels of insulin, insulin-like growth factors, and pros-taglandin ratios; lowered bile acid secretion; and altered gut flora 5. Moreover, there is evidence that colon cancer survivors experience significant declines in pa during adjuvant therapy 6 and that they report among the lowest pa participation rates of any cancer survivor group 7, suggesting that, in the current pa levels of colon cancer survivors, there is considerable room for improvement from a public health perspective. Finally, progress has been made in the science of health behaviour change over the past decade demonstrating that pa can, indeed, be increased substantially and maintained over extended periods of time with an appropriate behavioural support program 8–10. Together, these observations suggest that interventions to increase pa in colon cancer survivors may improve disease outcomes, that associated correlative biologic studies may provide insights into the mechanisms of colon cancer pathogenesis, and that sufficient understanding exists to implement an effective intervention.

Despite the highly suggestive observational findings, a randomized controlled trial (rct) is needed to establish unequivocally the causal nature of this association and to inform policies for health care delivery. The primary objective of the Colon Health and LifeLong Exercise Change (challenge) trial co.21 being undertaken by the National Cancer Institute of Canada Clinical Trials Group (ncic ctg) is to determine the effects of a 3-year structured pa intervention on dfs in survivors of high-risk stage ii or iii colon cancer who have completed adjuvant chemotherapy in the preceding 2–6 months and who are insufficiently active. Secondary objectives are to

- determine the effects of the pa intervention on important secondary endpoints including overall survival, multiple patient-reported outcomes (pros), and objective physical functioning.
- identify the determinants of long-term pa adherence in the intervention arm.
- explore the associations between selected molecular markers and study endpoint measures.
- provide an economic evaluation of the pa intervention.

We hypothesize that colon cancer survivors randomized to the pa intervention arm will experience improvements in dfs, pros, and objective physical functioning as compared with survivors allocated to general health education.

## 2. METHODS

### 2.1 Study Design and Participants

**FIGURE 1:**
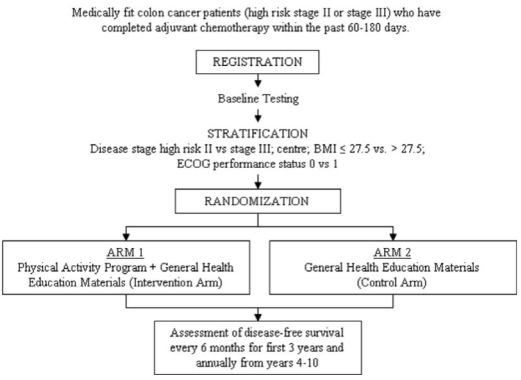
Flow of participants through the Colon Health and Life-Long Exercise Change (CHALLENGE) trial.

We will be conducting a multinational, multicentre, phase iii trial with randomization to a pa intervention versus general health education materials. This study will be led by the ncic ctg and will include collaboration with the Australasian Gastro-Intestinal Trials Group. Figure 1 presents the anticipated flow of participants through the trial. Medically-fit survivors of high-risk stage ii or iii colon cancer who have received their last dose of adjuvant chemotherapy within the preceding 60–180 days will be recruited. This group has been identified as being at high risk for disease recurrence or death, being most likely to adhere to the intervention, and being individuals for whom the pa intervention is safe. Table I details the eligibility criteria.

Pre-registration evaluations will focus on current pa involvement and medical eligibility for study participation. Patients who are already meeting current public health guidelines for pa (150 minutes or more of moderate-to-vigorous or 75 minutes or more of vigorous pa weekly), as estimated for the month before registration using the Leisure Time Exercise Questionnaire, will be ineligible. Consenting patients will be registered and will then undergo submaximal exercise testing to ensure that they are able to exercise safely at a moderate-to-vigorous intensity. Provided that 2 stages of the treadmill test are completed with acceptable heart rate and blood pressure responses, patients will then complete the remaining baseline investigations, including a physical functioning test, anthropometric testing, blood collection for correlative studies, pros, pa behaviour and determinants, health utility, and a health economics work productivity and activity impairment questionnaire (wpai). Patients will then be stratified by centre, disease stage (high-risk ii vs. iii), bmi (≤27.5 vs. >27.5), and Eastern Cooperative Oncology Group performance status (0 vs. 1), and will be randomized to receive the pa intervention program (intervention arm) or general health education materials (comparison arm).

### 2.2 Physical Activity Intervention

Participants in both arms will be provided with general health education materials including information about nutrition and pa. Participants in both groups will also receive follow-up care at the participating cancer centre, including regular physician visits, imaging, blood work, and colonoscopy. Participants assigned to the pa intervention will receive a structured 3-year pa program delivered by a local pa consultant (pac). The goal for the pa intervention group is to increase recreational pa from baseline by at least 10 met–hours weekly to a maximum of 27 met–hours weekly. The 10 met–hours weekly is roughly equivalent to the current public health guidelines of about 2.5 hours (for example, 5 days of 30 minutes daily) of moderate-intensity pa weekly, such as brisk walking (4 mets), or 1.25 hours (for example, 3 days of 25 minutes daily) of vigorous-intensity pa weekly such as jogging (8 mets). At the end of 6 months, a decision to encourage participants to increase pa by more than 10 met–hours weekly will be determined and will depend on their adaptation to the initial goal of an increase of 10 met–hours weekly. Patients will not be encouraged to increase their pa beyond 27 met–hours weekly, which is equivalent to about 1 hour daily of brisk walking for 7 days each week or 1 hour daily of jogging for 3.5 days each week.

Achieving an increase in pa from baseline of at least 10 met–hours weekly will require a significant amount of behaviour support. Participants in the intervention arm will therefore receive an intensive behaviour support program based on the Theory of Planned Behaviour 11 and modelled after the successful behavioural change program in the Diabetes Prevention Program 10 and the Look ahead trial 8. The pa intervention will be delivered in 3 distinct phases and will consist of behaviour support sessions and supervised pa sessions (Table II). The intervention will include a personalized pa prescription that accounts for the individual’s baseline fitness test results, pa history, performance status, personal preferences, and individual barriers to activity.

### 2.3 Behaviour Support Sessions

Behaviour support sessions will include training in behavioural strategies to promote the adoption and long-term maintenance of pa. Key behavioural strategies will include an emphasis on the unique benefits of pa for colon cancer survivors; strategies for making pa enjoyable, for overcoming barriers, for securing social support from family and friends, and for identifying environmental opportunities; and time management, self-monitoring, goal setting, planning, stimulus control, and self-reinforcement. At the core of the behavioural intervention will be a pa guidebook that will contain topics and materials that the pacs can use to reinforce and expand on during counselling sessions. The guidebook, called *Step Up to the Challenge!,* will be distributed to each pa intervention participant as an ongoing resource. It is modeled after a guidebook originally developed for breast cancer survivors and has been shown to be effective for increasing motivation, pa, and quality of life in breast cancer survivors 12–14. Throughout all phases of the study, pacs are able to provide additional behaviour support sessions if they determine that a patient is experiencing difficulty with adherence.

Advanced behavioural strategies will be considered for patients who have significant struggles in adopting or maintaining pa despite continued support from the pac. A behavioural toolbox will assist in overcoming barriers to pa, many of which will likely be related to issues of opportunity to perform exercise in the winter. The primary approach will be to have a facility available free of charge year round at each centre. All patients will also be provided with pedometers to motivate them and to help them track their pa.

### 2.4 Supervised Physical Activity Sessions

In addition to counselling sessions focusing on behaviour support, patients will also receive supervised pa sessions. These sessions will be combined with behaviour support sessions and will also occur independently. Their focus will be to teach proper pa technique and how to monitor intensity and to progress pa safely and effectively. In the first 6 months, these mandatory sessions will be supervised by the pac. Thereafter, supervision is strongly recommended. The supervision may be 1:1 with the pac or in a group format. Throughout all phases of the study, pacs will be able to provide additional supervised pa sessions if they determine that a patient is experiencing difficulties with adherence. Patients will be provided free access to a fitness facility at times outside of their supervised pa sessions.

### 2.5 Primary and Secondary Endpoints

Table III presents the nature and timing of the study evaluations. The primary endpoint is dfs, because available data demonstrate that 3-year dfs is highly correlated with overall survival in patients with resected colon cancer 15. This endpoint is now accepted for registration of trials by the U.S. Food and Drug Administration. Secondary endpoints will include overall survival, pros assessing quality of life [the Short Form (sf-36) and Functional Assessment of Cancer Therapy, General subscale], fatigue (Functional Assessment of Cancer Therapy, Fatigue subscale), sleep quality (Pittsburgh Sleep Quality Index), and anxiety and depression (Hospital Anxiety and Depression Scale), and objective testing of physical functioning consisting of anthropometric measurements, cardiovascular fitness (submaximal exercise test using the Balke treadmill protocol 16); and physical functioning (Seniors Fitness Test 17). Adherence to pa will be assessed at baseline and every 6–12 months by using a slight modification of the Past Year Total Physical Activity Questionnaire 18,19. The Past Year Total Physical Activity Questionnaire has been shown to have acceptable reliability and validity for measurement of past-year pa 19. Correlative biologic markers will include blood measures of insulin, insulin-like growth factors, and selected cytokines. Finally, an economic evaluation will be performed to assess cost-effectiveness and cost–utility of the pa intervention. Patient utilities will be measured using the sf-6D, which is derived from the sf-36 quality-of-life measure 20 that has been validated in population surveys and clinical trials settings. Resource utilization and wpai will be collected prospectively as part of the economic analysis.

### 2.6 Statistical Considerations

The analysis will be performed using the intent-to-treat principle and is powered to detect a hr of 0.75 for dfs between patients randomized to two treatment arms. To detect such a hr with a power of 80% and a two-tailed alpha of 0.05, we will need to observe 380 events during follow-up. It is anticipated that a total of 962 patients will be randomized over 3 years. To observe 380 events, we assume that the patients in the comparison group will have a 3-year dfs of 75% and will not materially change their pa behaviour from baseline. It is also assumed that 20% of patients randomized to the intervention group will not adhere to their pa program. With these assumptions, it is estimated that, to observe 380 events among 962 patients randomized, 4.7 years of additional follow-up after the last patient is randomized will be required, which leads to an approximate total study duration of 7.7 years. Four interim analyses will be performed: the first analysis will assess the feasibility of accrual principles, the second will assess the feasibility of the pa behaviour change, and the final two will assess for unexpectedly large magnitudes of benefit or futility.

### 2.7 Trial Management

The development and oversight of the co.21 trial is provided by the Trial Steering Committee, which includes the study chairs and the Design and Conduct Committee members; the coordinators for Quality of Life, Economic Evaluations, and Correlative Sciences committees; and the ncic ctg central office physician coordinator, senior biostatistician, and study coordinator. Because of the complexities of delivering a lifestyle intervention and because of the uniqueness of this study, four working groups were established to provide expertise in protocol development and trial implementation in four key areas: pa, pros, health economics, and correlative studies. Each of these working groups is chaired by a member of the Trial Steering Committee with expertise in the respective field. A PA Working Group will provide oversight to the pa and behaviour support components of the study, including monitoring of adherence. This working group is led by the study chair.

## 3. DISCUSSION

Colorectal cancer is the third most common cancer in Canadian men and women and the second leading cause of cancer-related death 21. There are more than 110,000 colorectal cancer survivors in Canada. This growing number of survivors has generated interest in behaviour and lifestyle interventions that might further improve disease outcomes and quality of life. In colon cancer, pa has been strongly associated with improved disease outcomes, and there are plausible biologic mechanisms for this association. However, the data published to date have been observational. Moreover, few colon cancer survivors currently exercise, partly because of the physical and psychological effects of their disease and treatments, and partly because of the lack of guidance and support from cancer care organizations. However, recent progress in behavioural change suggests that, with appropriate support, such populations can be motivated to adopt long-term lifestyle changes. Consequently, all the necessary evidence, rationale, and justification exist to warrant the conduct of an rct of pa and disease outcomes in colon cancer survivors.

Moreover, in the era of molecularly targeted anti-cancer therapy, funding of new and expensive agents is becoming increasingly difficult. Should a pa intervention be found to have a significant clinical benefit in colon cancer patients, this type of intervention could be a very cost-effective therapy, with the potential for many other non-cancer-related health benefits. The magnitude of the associations between pa and disease outcomes in the observational studies of colon cancer compares favourably with the benefit observed with the use of adjuvant chemotherapy, but would likely involve lower toxicity and cost. Importantly, the cost-effectiveness of this pa intervention will be prospectively evaluated as part of this clinical trial, because all interventions are recognized to have a cost component that needs to be considered when evaluating the effectiveness of the intervention to reduce the burden of a disease at a population level.

Positive findings from the challenge trial would support a new and additional paradigm to be tested in patients with other forms of cancer and would enhance interest in applying these results to test strategies for cancer prevention. One of the greatest challenges to implementing pa programs in any population has been ensuring adequate support and resources to promote behaviour change with regard to pa. Cancer patients are, in general, a sedentary but motivated population. Previous work has suggested good adherence and a willingness to pursue lifestyle modifications following a diagnosis of cancer. If this rct is able to demonstrate a significant benefit in dfs, that finding would provide an impetus to patients to participate in pa and to oncologists and cancer care organizations to promote pa. It could also potentially lead to changes in public perception that might influence the policies by which pa experts are utilized within the health care system. The challenge trial will also have an opportunity to evaluate the cost-effectiveness of potential policy recommendations.

## 4. CONCLUSIONS

In various populations, both healthy and with a disease, pa has been associated with numerous health benefits. As yet, no conclusive evidence has been found that pa will reduce the likelihood of colon cancer recurrence or will extend survival. Our study has the potential to answer this question definitively and will provide valuable insights into this association regardless of the final results of the trial. In addition, the study will obtain robust data about the effect of pa on important secondary endpoints in colon cancer survivors including quality of life, fatigue, mood, biologic correlative measures, and cost-effectiveness.

## Figures and Tables

**TABLE I:** Inclusion and exclusion criteria for the challenge trial

Inclusion criteria for registration
Completely resected, histologically documented, high-risk stage ii or iii adenocarcinoma of the colon Adjuvant chemotherapy treatment for colon cancer with a 5-fluorouracil–based regimen given with an intent to provide 24 weeks of treatment (actual treatment may be less than 24 weeks, and patients must have received a minimum of 1 treatment cycle) Chemotherapy completed 60–180 days before registration 18 Years of age or older Medically suitable for exercise testing and for participation in physical activity (by revised Physical Activity Readiness Question naire and investigator opinion) Eastern Cooperative Oncology Group performance status of 0 or 1 Adequate hematologic, renal, and hepatic function No evidence of metastatic or locally-recurrent colon cancer [by chest radiography or computed tomography (ct), and ct of abdomen and pelvis] Current physical activity levels that do not meet the current recommended guidelines (150 minutes or more of moderate-to- vigorous or 75 minutes or more of vigorous physical activity weekly) calculated using the Godin–Shephard Leisure Time Exercise
Questionnaire
Ability and willingness to effectively communicate with the physical activity consultant Ability and willingness to complete all questionnaires involved in study (provided in English and French) Informed consent granted Accessibility for treatment and follow-up
Exclusion criteria for registration
Significant comorbid conditions precluding participation in a physical activity program Locoregional or distant metastatic disease Unlikely to participate in a physical activity program (as assessed by the investigator) History of other malignancies, except adequately treated non-melanoma skin cancer; curatively treated *in situ* cancer of the cervix or other solid tumours; Hodgkin lymphoma or non-Hodgkin lymphoma curatively treated, with no evidence of disease for more than 5 years Treatment with a beta-blocker and unwillingness or inability to discontinue that medication for 48 hours before each required exercise test, or any other medications deemed by the investigator to be likely to preclude participation in a physical activity program Current treatment with additional chemotherapy or radiation Inability to complete the baseline exercise test Current pregnancy or plans to become pregnant within the next 3 years
Inclusion criteria for randomization
Completion of at least 2 stages of the submaximal exercise test with acceptable heart rate and blood pressure response Completion of anthropometric testing, a Senior’s Fitness Test, Patient Reported Outcomes, Health Economics, and Physical Activity Behaviour and Adherence questionnaires (including the Total Physical Activity Questionnaire to calculate baseline weekly metabolic equivalent task–hours) Mandatory blood samples for correlative studies Fasting glucose sample Protocol intervention to begin within 14 days of patient randomization

**TABLE II:** Physical activity program intervention

Content	Baseline to 6 mo.	Phase 6–12 mo.	12–36 mo.
Behaviour support sessions a	12 Mandatory face-to-face sessions held biweekly	12 Mandatory sessions held biweekly, with option for face-to-face or telephone delivery	Mandatory monthly sessions, with option for face-to-face or telephone delivery
Supervised physical activity sessions a,b	12 Mandatory sessions combined with the mandatory behaviour support sessions 12 Additional supervised physical activity sessions on alternate weeks strongly recommended	12 Sessions recommended; can be combined with the biweekly behaviour support sessions for those who choose face-to-face sessions	Monthly sessions recommended; can be combined with the monthly behaviour support sessions for those who choose face- to-face sessions
Physical activity goal c	Gradually increase recreational physical activity by 10 metabolic equivalent task (met)– hours weekly over baseline (to 10–19 met– hours weekly)	Individualized (based on phase i results) to a maximum increase of 20 met–hours weekly (to a total of 20–27 met–hours weekly)	Individualized (based on phase ii results) to a maximum total of 27 met– hours weekly

a Physical activity consultants can provide additional behavioural support and supervised physical activity sessions in any phase if they determine that a patient is experiencing difficulty with adherence.

b Patients will be provided with access to a fitness facility outside of the scheduled sessions; however, no behaviour support or physical activity supervision will be provided.

c All increases in physical activity refer only to recreational activity metabolic equivalent task (met)–hours weekly.

**TABLE III:** Study evaluations

Required investigations	Before		Until recurrence or new primary malignancy			After recurrence or new primary malignancy		
	Reg.	Rand.	Every 6 mo. (years 1–3)	Every 12 mo. (years 4–5)	Every 12 mo. (years 6–10)	Every 6 mo. (years 1–3)	Every 12 mo. (years 4–5)	Every 12 mo. (years 6–10)
History and physical								
Blood pressure + heart rate	X							
History, physical exam, weight, height	X		X	X	X	X	X	X
Disease status and overall survival	X		X	X	X	X	X	X
ecog performance status	X							
Exercise screening questionnaire	X							
Medical Suitability for Exercise questionnaire	X							
Major medical problems	X							
Concomitant medications	X		X	X	X	X	X	X
Hematology								
Absolute granulocyte count + hemoglobin + platelets + white blood cells	X							
Biochemistry								
Liver function tests (bilirubin, alkaline phosphatase, ast or alt)	X							
Creatinine	X							
Carcinoembryonic antigen	X		X	X				
Radiology								
Chest radiography or computed tomography	X		X	X				
Abdomen and pelvis computed tomography	X		X	X				
Colonoscopy			Once, 30–48 mo. after randomization					
Other			As clinically indicated to document recurrence or new primary malignancy					
Fitness testing								
Submaximal Exercise Test		X	X^a^					
Seniors’ Fitness Test		X	X^a^					
Hip and waist circumference		X	X^a^					
Other investigations								
Serum and plasma		X		X^b^				
Fasting glucose		X						
Adverse events								
Adverse event assessments	X		X	X	X	X	X	X
Patient-reported outcomes								
fact-F		X	X	X				
sf-36 (quality of life)		X	X	X		X	X	
Pittsburgh Sleep Quality Index		X	X	X				
Hospital Anxiety and Depression Scale		X	X	X				
Physical activity behaviour								
Total Physical Activity Questionnaire		X	X	X				
Social-Cognitive Determinants of Exercise		X	X					
Health economics								
Resource Utilization Assessment			X	X		X	X	
Work Place Activity and Impairment Questionnaire		X	X	X		X	X	
30-Day Resource Use Diary			X^c^	X		X	X	

a At 6, 12, 24, and 36 months.

b At 12, 24, and 36 months.

c Including the first month of the intervention period.

Reg = registration; Rand = randomization; ecog = Eastern Cooperative Oncology Group; ast = aspartate aminotransferase; alt = alanine aminotransferase; fact-F = Functional Assessment of Cancer Therapy, Fatigue subscale; sf-36 = Short Form 36.
